# The role of interleukin-1 in general pathology

**DOI:** 10.1186/s41232-019-0101-5

**Published:** 2019-06-06

**Authors:** Naoe Kaneko, Mie Kurata, Toshihiro Yamamoto, Shinnosuke Morikawa, Junya Masumoto

**Affiliations:** Department of Pathology, Ehime University Graduate School of Medicine and Proteo-Science Center, Shitsukawa 454, Toon, Ehime 791-0295 Japan

**Keywords:** Interleukin-1, Metabolic inflammation, Inflammatory diseases, Cancer, Drug target

## Abstract

Interleukin-1, an inflammatory cytokine, is considered to have diverse physiological functions and pathological significances and play an important role in health and disease. In this decade, interleukin-1 family members have been expanding and evidence is accumulating that highlights the importance of interleukin-1 in linking innate immunity with a broad spectrum of diseases beyond inflammatory diseases. In this review, we look back on the definition of “inflammation” in traditional general pathology and discuss new insights into interleukin-1 in view of its history and the molecular bases of diseases, as well as current progress in therapeutics.

## Background

In terms of general pathology, inflammation is one of the adaptive responses to various injuries including physical, chemical, and biological factors. The Roman encyclopedist A. Cornelius Celsus described four cardinal signs of inflammation in one concise sentence: “Now the signs of an inflammation are four: redness (rubour) and swelling (tumour), with heat (calour) and pain (dolour)” [1]. A century and a half later, Galen added a fifth sign: “disturbance of function” (funcio laesa) [2]. The classical signs of inflammation are considered to be related to cells and tissues responding to pathological cell injury caused by internal stimuli, including damage-associated products and metabolites, and external stimuli, including bacteria and viruses [3–6].

The host bears the receptors that facilitate recognition of these damage-associated molecular patterns (DAMPs) and/or pathogen-associated molecular patterns (PAMPs) that are not host-derived. These receptors are termed pattern recognition receptors (PRRs) [7]. PRRs directly or indirectly detect infection and/or noxious chemicals, resulting in inflammation that is coupled with the induction of immune responses and a tissue reparative component [8]. The signal transduction triggered by these PRRs leads to the acute inflammatory mediator expressions that regulate the elimination of pathogens and infected cells [9, 10].

There are several known PRRs: Toll-like receptors (TLRs), RIG-I-like receptors (RLRs), NOD-like receptors (NLRs), and C-type lectin receptors (CLRs). The majority of NOD-like receptors such as NLRP1, NLRP3, NLRC4, NLRP6, and NLRP12 can interact with apoptosis-associated speck-like protein containing a caspase-recruitment domain (ASC) and caspase-1, and the resulting complex is a sensor of cell injury called “inflammasome”, an interleukin (IL)-1β-processing platform that plays a crucial role in IL-1β maturation and secretion from cells. Other pyrin-domain (PYD)-containing proteins such as AIM2, IFI-16, and pyrin are also known to form inflammasomes. Among them, NLRP3 inflammasomes monitor membrane integrity and pore-forming toxins, crystals, and many other noxious stimuli and are involved in IL-1β processing and maturation [11–14]. It is now widely accepted that an inflammatory response is the extreme end of a spectrum that ranges from a homeostatic state of inflammation to a stress response and finally inflammation [8, 15]. The homeostatic state of inflammation, which is not inflammation from the perspective of general pathology, was suggested to be maintained by PRRs expressed in stromal and/or immune cells, detecting endogenous ligands in parenchymal cells and/or pathogens, leading to chronic inflammatory responses ranging from the basal homeostatic state to disease-causing inflammation [15, 16]. In addition to several forms of inflammation including classical inflammation, homeostatic inflammation, a distinct nomenclature for low-grade inflammation, such as para-inflammation (an adaptive response against stress or malfunction) and meta-inflammation (metabolically triggered inflammation), has been proposed [17–19]. As discussed above, there are various factors involved in forms of inflammation; in particular, since IL-1 is a downstream cytokine of the sensor of cell injury, the inflammasome, it is important for regulating inflammation and tissue damage beyond inflammation [20].

## Biological functions of interleukin-1

IL-1 is a master regulator of inflammation via controlling a variety of innate immune processes [21]. From a historical point of view, IL-1 has a wide range of biological functions, which include acting as a leukocytic pyrogen, a mediator of fever and a leukocytic endogenous mediator, and an inducer of several components of the acute-phase response and lymphocyte-activating factor (LAF) [22, 23]. LAF was later shown to be a macrophage-derived immune mediator acting on T- and B- lymphocytes and was designated as IL-1 in the Second International Lymphokine Workshop held in Switzerland in 1979 [24, 25]. In addition, serum blocking factors in breast cancer patients identified by the leukocyte adherence inhibition test were reported. The serum adherence-promoting factors were regulated by IL-1 [26–28]. To date, the tumor microenvironment has been characterized by dominant immunosuppression, being infiltrated by tumor immunosuppressive myeloid-derived suppressor cells (MDSCs), regulatory T cells (Tregs), and tumor-associated macrophages (TAMs) [29, 30]. IL-1 is capable of inducing the recruitment of TAMs and MDSCs, which promote tumor development in breast cancer [31].

### Interleukin-1 family members

Currently, human sequence algorithm technologies suggest that the IL-1 family comprises a total of 11 members with similar or distinct biological effects [32, 33]. IL-1α, IL-1β, IL-1Ra, IL-18, IL-33, IL-36α, IL-36β, IL-36γ, IL-36Ra IL-37, and IL-38 have been identified and characterized (Table 1) [32]. Among them, IL-1α, IL-1β, IL-18, IL-33, and IL-36 are receptor-agonistic, and IL-1Ra, IL-36Ra, and IL-38 are receptor-antagonistic. IL-37 is the only anti-inflammatory cytokine. Although the function of each IL-1 family member is now being investigated, IL-1 is the most characterized among these members.

**Table 1:** The IL-1 family members

IL-1 family members	Receptor	Property
IL-1α	IL-1RI	Inflammatory
IL-1β	IL-1RI	Inflammatory
IL-1Ra	IL-1RI	IL-1RI antagonist
IL-18	IL-18Rα	Inflammatory
IL-33	ST2	Th2 inflammation
IL-36Ra	IL-1Rrp2	IL-1Rrp2 antagonist
IL-36α	IL-1Rrp2	Inflammatory
IL-36β	IL-1Rrp2	Inflammatory
IL-36γ	IL-1Rrp2	Inflammatory
IL-37	IL-18Rα	Anti-inflammatory
IL-38	IL-1Rrp2	IL-1Rrp2 antagonist

### Molecular mechanism of interleukin-1 activation

There are two individual forms of IL-1, IL-1α and IL-1β, isolated from two distinct cDNAs, but they are indistinguishable in terms of their biological functions [34]. Although the homology between IL-1α and IL-1β is not high (27%) in terms of amino acid sequences, IL-1α and IL-1β are structurally similar and show the same functions by sharing a common receptor, IL-1 type 1 receptor (IL-1R1), and both have the same central β-barrel along with adjoining loops [35, 36]. The difference between IL-1α and IL-1β is an N-terminal extension of 14 residues beyond the N-terminus of IL-1α and IL-1β [37]. The molecular weight of each precursor is approximately 31 kDa, and IL-1α and IL-1β are processed by specific proteases to mature forms. The N-terminal domain of IL-1α contains a nuclear localization sequence (NLS) and shows transcription activity [38]. IL-1α is produced as a 271-amino acid (AA) precursor protein. For transcription of the IL-1α gene, transcription factor specificity protein 1 (Sp1) activates the IL-1α promoter activity in the 5′-upstream GC box (− 60 to − 45 bp) [39] and NF-κB, which is also activated by IL-1α itself, and stimulates the consensus promoter region (− 103 to − 70 bp) to induce its own gene expression and production in an autocrine manner [40]. The precursor of IL-1α translocates into the nucleus to bind to chromatin and also exists in a membrane-anchored form. Upon stress responses, IL-1α is processed by Ca^2+^-dependent protease calpain or other proteases, such as cytotoxic T- lymphocytes (CTL)/natural killer (NK)-granzyme-B, mast cell chymase, or neutrophil elastase to the C-terminal 159 AA as mature IL-1α [41]. The IL-1α processing separates NLS from its precursor, which is not linked to secretion or cell death [21]; however, IL-1α is a key danger signal that induces inflammation on release from necrotic cells [42]. The IL-1α precursor triggers IL-1R1 on resident macrophages in necrotic tissues, producing IL-1β as well as chemokines as post-necrotic inflammation [43].

IL-1β is produced as a 269-AA precursor protein and processed by caspase-1, which is also known as IL-1β-converting enzyme (ICE), activated in inflammasomes, to the C-terminal 153 AA as mature IL-1β [11, 12, 34, 44]. The IL-1β precursor is also processed by other serine proteases [45]. Neutrophils derived from caspase-1-deficient mice release mature IL-1β processed by elastase in response to lipopolysaccharide (LPS) stimulation [46]. The neutrophil proteases, such as elastase, chymases, granzyme A, cathepsin G, and proteinase-3, cleave the IL-1β precursor into a secreted, biologically active form [47–49]. These alternatively cleaved forms of functional IL-1β were detected in synovial fluid of a patient with inflammatory polyarthritis and gout [50]. Occasionally, massive neutrophil infiltration appeared in excess-inflammation-damaged tissues and organs, such as in septic shock or systemic inflammatory response syndrome. Thus, the NLRP3 inflammasome-related inflammation induced by a variety of factors described above may be a target of anti-IL-1 therapy [51].

Currently, a two-step model of the initiation of NLRP3 inflammasome activation is suggested. The first step mediates transcriptional and post-translational priming of NLRP3 (Step1), and the second step is activation of inflammasomes (Step 2). Step 1 is the first synthesis of a biologically inactive IL-1β precursor by NF-κB binding to the consensus binding site (− 296 to − 286 bp) to transcribe the IL-1β gene. Step 2 is processing into mature, biologically active IL-1β by caspase-1 activated by a cytosolic activation platform called inflammasome [52, 53]. The inflammasome is a large protein complex, which consists of PRRs, such as NLRs, AIM2, RIG-I or pyrin, an adaptor protein ASC, and caspase-1. Among them, the NLRP3 inflammasome is a prototype inflammasome, which has been reported to be activated by a wide range of PAMPs and DAMPs [54, 55]. Various NLRP3-activating PAMPs have been reported, i.e., bacteria-derived RNA or DNA, pore-forming toxins, lethal toxins, flagellin/rod proteins, muramyl dipeptide (MDP), M2 protein, virus-derived RNA or DNA, fungus-derived β-glucans, hypha mannan, zymosan, and protozoon-derived hemozoin [56]. NLRP3-activating DAMPs have also been reported, i.e., self-derived glucose, β-amyloid, hyaluronan, ATP, cholesterol crystals, monosodium urate (MSU) crystals, calcium pyrophosphate dihydrate (CPPD) crystals, environment-derived alum, asbestos, silica, alloy particles, UV radiation, and skin irritants [56]; however, their diverse physiological and chemical signals leading to the direct activation of NLRP3 have not been fully elucidated. Instead, efflux of potassium has been identified as the common activator of most known NLRP3 step 2 signals [57, 58]. The NLRP3 activation by potassium efflux suggested to lead NLRP3-Nek7 interaction to drive inflammasome activation [59–61]. The mechanism underlying the secretion of IL-1β has been suggested to overlap with IL-1α secretion [41]. Also, mitochondrial and phagosomal reactive oxygen species (ROS) have been proposed to activate the NLRP3 inflammasome. Alternatively, non-canonical pathways of NLRP3-inflammasome activation associated with proinflammatory caspases, caspase-4, caspase-5, and caspase-11 have been proposed. In this process, LPS is recognized by the caspase-recruitment domain (CARD) of respective caspases, leading to activation [62–65]. Caspase-8 or proteases in neutrophils are also processed and activate IL-1β. Several PRRs, such as NLRP1, NLRP3, NLRC4, pyrin, and AIM2, convert the assembly of the adaptor molecule ASC into a high-molecular-weight complex, called the pyroptosome [66]. Then, the caspase-1 precursor is recruited to the pyroptosome to also form helical structures, which enable its proximity-induced proteolytical auto-activation. With caspase-1 precursor maturation into the active p102/p202 heterotetramer, it cleaves the IL-1β precursor, leading to pyroptotic cell death. This cell death is mediated by the caspase-1-dependent cleavage of gasdermin-D (GSDMD) [67–69]. In turn, the mature N-terminal fragment of GSDMD translocates to the inner leaflet of the plasma membrane to form round and pore-like structures of approximately 15 nm in diameter [70–73].

### Tissue distributions of interleukin-1

IL-1α and IL-1β are expressed in a wide range of tissues and a variety of cells, especially in macrophages in lymphoid organs including the thymus, spleen, lymph nodes, Peyer’s patches, and bone marrow. In non-lymphoid organs, IL-1α and IL-1β are expressed in tissue macrophages in the lung, digestive tract, and liver. They are also expressed in cellular subepithelial endometrial tissue of the uterus, in the glomeruli, in outer cortical areas of the kidney, and in various specific cell types, including neutrophils, keratinocytes, epithelial and endothelial cells, lymphocytes, smooth muscle cells, and fibroblasts [74, 75].

### Interleukin-1 receptors and subcellular signaling

There are two cell surface IL-1 receptors, IL-1R1 and IL-1 type 2 receptor (IL-1R2), a decoy receptor. IL-1 binds to IL-1R1, which requires the formation of a heterodimer with the IL-1 type 3 receptor (IL-1R3) (also known as IL-1RAcP) accompanied by adaptor IL-1 receptor-associated kinase (IRAK) and myeloid differentiation primary response protein 88 (MyD88) [76]. IL-1R1 initiates inflammatory responses when binding to the ligands IL-1α and IL-1β and has been reported to be expressed by T- lymphocytes, fibroblasts, epithelial cells, and endothelial cells. IL-1R2, which does not initiate signal transduction, is expressed in a variety of hematopoietic cells, especially in B- lymphocytes, mononuclear phagocytes, polymorphonuclear leukocytes, and bone marrow cells. Notably, expression levels of IL-1R1 and IL-1R2 are different among the cell types; for example, neutrophils predominantly express IL-1R2. As a result, much higher concentrations of IL-1β are required to activate neutrophils, whereas low concentrations of IL-1β are sufficient to activate endothelial cells. The IL-1R1-mediated signaling pathways also differ according to the cell types [77, 78]. IL-1R3 is a co-receptor for IL-1R1, responsible for signaling after binding ligands IL-1α and IL-1β, and has been reported to be ubiquitously expressed by all cells responsive to IL-1. IL-1R3b is a brain-specific isoform of IL-1R3 generated by alternative splicing, and it has been reported to be expressed in the brain, cerebellum, and spinal cord [79].

Activated IL-1 is incapable of functioning until recognized by cell surface receptors. The complex contains a motif of GTPase activity and activates GTPase-activating protein and protein kinases [80–82]. In contrast, IL-1R2 is thought to reduce the biological response to IL-1. The proximity of the two cytoplasmic domains of IL-1R1 and IL-1R3 is thought to initiate signal transduction by the hydrolysis of GTP. This is followed by c-Jun N-terminal kinase (JNK) and p38 MAP kinase [83]. IRAK and tumor necrosis factor (TNF) receptor-associated factor (TRAF) 6 activate NF-κB, as well as p38, JNKs, extracellular signal-regulated kinases (ERKs), and mitogen-activated protein kinases (MAPKs) [84]. The NF-κB activation pathway is dependent on the Iκ-B kinase (IKK) complex, composed of IKKα, IKKβ, and NF-κB essential modulator (NEMO), via associations with TAK1, TAK2, TRAF2, and TRAF6 in the IL-1R1-signaling pathway [85]. These signals play important roles in both acute and chronic inflammation in various diseases [86].

### Interleukin-1 and diseases

#### Autoinflammatory diseases

Single nucleotide mutation of the *CIAS1* gene results in NLRP3 mutation, which induces constituted inflammasome activation causing cryopyrin-associated periodic syndrome (CAPS). This is termed autoinflammatory disease, including familial cold autoinflammatory syndrome (FCAS), Muckle–Wells syndrome (MWS), and neonatal-onset multisystem inflammatory disease (NOMID)/chronic infantile neurologic, cutaneous, and arthritis (CINCA) syndrome, which leads to greater IL-1β secretion without any DAMPs or PAMPs [87–92]. The most common autoinflammatory disease is Familial Mediterranean fever (FMF). FMF is autosomal recessive; however, mutations in the causative *MEFV* gene, encoding mutated pyrin, leads to active pyrin inflammasome [93]. Inflammatory diseases like those above, characterized by the enhanced secretion of IL-1β, include a group of autoinflammatory diseases such as NLRP12 autoinflammatory syndrome; hyperimmunoglobulinemia D and periodic fever syndrome (HIDS)/mevalonate kinase deficiency (MKD); pyogenic arthritis, pyoderma gangrenosum, and acne (PAPA) syndrome; pyoderma gangrenosum, acne, and suppurative hidradenitis (PASH) syndrome; pyogenic arthritis, acne, pyoderma gangrenosum, and suppurative hidradenitis (PAPASH); Majeed syndrome; and TNF-receptor-1-associated syndrome (TRAPS) [93–100]. On deficiency of the IL-1-receptor antagonist (DIRA), excess IL-1β induces other proinflammatory cytokines and chemokines [101].

#### Metabolic syndromes

Excess stress responses disrupt body homeostasis under physiological conditions and lead to excess cytokine production. NLRP3 inflammasomes have also been reported to be involved in low-grade subclinical inflammation induced by chronic exposure to high levels of free fatty acids and glucose, leading to increased apoptosis and impaired insulin secretion of β-cells in obese type 2 diabetes mellitus (T2D) patients [102–104]. Indeed, islet amyloid polypeptide (IAPP) oligomers activated NLRP3 inflammasomes to induce significant IL-1β production by infiltrating macrophages in an in vivo study [105, 106]. Higher concentrations of glucose activate NF-κB and IL-1 precursors in cells [102]. Minimally oxidized low-density lipoproteins stimulate TLR4, which triggers IL-1β expression [104, 105], and accumulations of islet amyloid polypeptides are deposited and mediate NLRP3 inflammasome activation in islet macrophages [107]. Another oligomer of amyloid, amyloid β, can induce IL-1β via NLRP3 inflammasomes in a process involving the phagocytosis of amyloid β in glial cells in patients with Alzheimer’s disease (AD) and subsequent lysosomal damage and release of cathepsin B [108]. ROS are considered to be involved in the activation of NLRP3 inflammasomes, and it was suggested that direct interaction between amyloidogenic peptide and NLRP3 could initiate NLRP3 inflammasome formation in a cell-free system [109, 110]. Both IL-1α and IL-1β gene polymorphisms have been reported to be associated with central obesity and metabolic syndrome in a population with coronary heart disease in an epidemiologic study [111]. Thus, these diseases are IL-1-dependent cytokinopathies (interleukinoneopathies).

#### Acute inflammation

Besides the above diseases, numerous inflammatory diseases related to excess IL-1 signaling have also been identified [112–114]. For example, high IL-1β levels in humans and mice result in increased Th17-dominant immunopathology, and IL-1β expression was limited to macrophages and neutrophils, which account for a large proportion of the CD45α cells in the cervix upon *Chlamydia muridarum* infection [115]. Consequently, IL-1β promotes the differentiation of monocytes into conventional dendritic cells (DCs) and M1-like macrophages and supports the proliferation of activated B- lymphocytes and their differentiation into plasma cells [116–118]. IL-1 in combination with IL-2 promoted not only the expansion of NK cells but also CD4+ CD8+ T-lymphocytes [119]. IL-1β generated by activated antigen-presenting cells (APCs) induced type 1 immune responses, which produced CTL and led to the polarization of CD4+ T -lymphocytes towards T-helper cell type 1 (Th1) [120, 121].

#### Chronic inflammation and malignancy

IL-1β plays a role in resolving acute inflammation resulting in the initiation of adaptive anti-tumor responses; however, chronic inflammatory conditions increase the risk of developing cancer [122]. In human breast cancer, higher expression of IL-1β is associated with tumor invasiveness and aggressive tumor biology [123]. Expression of IL-1α, IL-1β, and their receptors in human breast cancer tissues results in the activation of a population of cells and subsequently contributes to angiogenesis, tumor proliferation, and tumor invasion in the microenvironment [124]. In a spontaneous MMTV-PyMT mouse mammary gland tumor model, mature IL-1β levels in primary mammary tumors and metastasis sites were significantly elevated, being associated with inflammasome activation and the infiltration of myeloid cells in tumor microenvironments. In this model, CD11b^+^Gr1^+^ and CD11b^+^Gr1^−^ myeloid cell populations were also significantly increased in both tumor tissues [31]. IL-1β generated in a tissue with a tumor microenvironment dominated by TAMs promotes tumor growth and metastasis in breast cancer [122, 125]. IL-1, by promoting MDSCs and sustaining the immunosuppressive activity of TAMs, contributes to the suppression of effective adaptive anti-tumor immune responses [126]. Actually, the sphingolipid sphingosine-1-phosphate (S1P) on TAMs promotes lymphangiogenesis and lung metastasis via NLRP3/IL-1β in mouse breast cancer model [127]. For example, obesity induces an increase in tumor-infiltrating MDSCs with activated NLRC4 inflammasome, leading to IL-1β production, which drives tumor progression through adipocyte-mediated vascular endothelial growth factor (VEGF) A expression and angiogenesis [128]. A recent report showed that IL-1β orchestrates tumor-promoting inflammation in patients with high-risk HER2-negative breast cancer who would benefit from IL-1-blocking therapeutics with anakinra (described later on). The report indicates that while anakinra downregulates gene expressions for IL-1β, IL-1R1, IL-1R2, and IL-1R3, increased gene expressions of NK cells and CTLs are observed [129].

## Interleukin-18 and diseases

Although IL-1 has been well-characterized, IL-18 and other IL-1 family members have been less comprehensively investigated. IL-18 can be processed by caspase-1 and proteinase-3 as well as IL-1β, to be activated [130–132]. Considering the pathogenesis of IL-1-related diseases, IL-18 could be involved [133].

IL-18 was originally identified as interferon (IFN)-γ-inducing factor [134]. IL-18 is the most structurally related to IL-1β. IL-18 is synthesized as a 24-kDa inactivated precursor and is cleaved by caspase-1 to a biologically active 17-kDa mature form [131, 132]. Although IL-1β is biologically active within the pg/mL range, IL-18 requires 10–20 ng/mL and sometimes higher levels for in vitro activation [135, 136]. Since the IL-18 precursor is expressed ubiquitously in tissues [137], IL-18 signaling is thought to be regulated concentration-dependently. Mature IL-18 forms a signaling complex with the IL-18 receptor alpha chain (IL-18Rα) with low affinity. If the cell expresses an IL-18 receptor β chain (IL-18Rβ), a high affinity complex is formed like the IL-1R accessory chain IL-1R3. The complex of the heterodimer recruits MyD88 through the Toll-IL-1 receptor (TIR), four IRAKs, and TRAF-6, leading to the degradation of I-κB and activation of NF-κB, as that for IL-1 signaling [83].

IL-18 is involved in regulation of the Th1 response by modulating the production of IFN-γ. For example, in synergy with either IL-12 or IL-15, which upregulates the expression of the IL-18Rβ co-receptor, IL-18 induces the production of IFN-γ by T cells [138]. IL-18 induces IFN-γ production by NK cells, and NK cells express CCR7 and produce high levels of IFN-γ [139]. The combination of IL-18 and IL-12 induced high levels of IFN-γ upon hypoglycemia, intestinal inflammation, and inanition [140]. Some human autoimmune diseases are associated with the elevated production of IFN-γ and IL-18. Autoimmune diseases such as systemic lupus erythematosus, rheumatoid arthritis (RA), type-1 diabetes mellitus, Crohn’s disease and psoriasis, and graft versus host disease are thought to be mediated by IL-18 [141]. So far, several anti-IL-18 therapies have been reported. An anti-IL-18, multicenter, randomized, single-blind, placebo-controlled, parallel-group, phase IIa trial for the treatment of T2D was reported whereby anti-IL-18 monoclonal antibody, GSK1070806, was well-tolerated; however, the anti-IL-18 therapy did not lead to any improvements in glucose control [142]. Interleukin-18 binding protein (IL-18BP) was purified from urine by chromatography on IL-18 beads that abolished IL-18 induction of IFN-γ, IL-8, and activation of NF-κB in vitro [143]. The IL-18 inhibition using IL-18BP significantly decreased MDSCs in the tumor microenvironment in a preclinical osteosarcoma mouse model [144]. IL-18BP (Tadekinig α®) was successful in the treatment of Still’s disease and NLRC4-mutated autoinflammatory macrophage activation syndrome (MAS), for which anti-IL-1 treatment had failed [145, 146].

### Biologics against interleukin-1 signaling and their applications

**Fig. 1:**
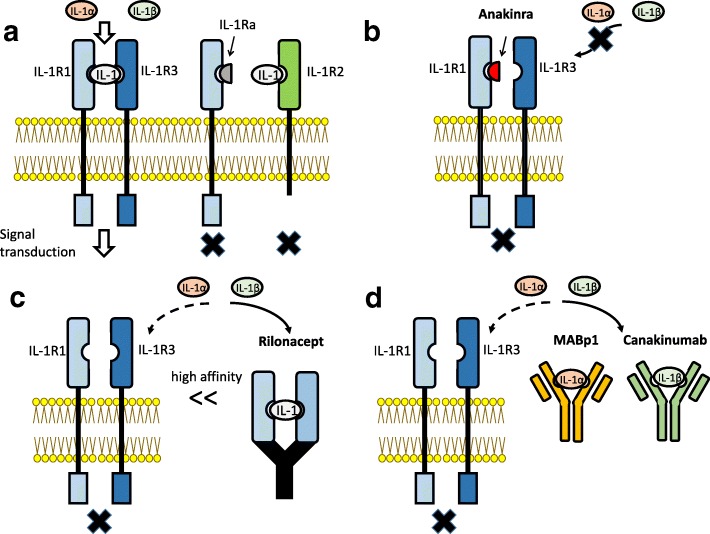
Interleukin-1 receptors and inhibitors of IL-1 signaling. **a** IL-1R1 interacts with both IL-1α and IL-1β and promotes signal transduction, together with its co-receptor IL-1R3 (IL-1RAcP). IL-1Ra is a protein that binds to IL-1R1 but not IL-1R3, and it is as an inhibitor of IL-1 signaling. IL-1R2 is a decoy receptor because it lacks a cytoplasmic segment. **b** Anakinra is a recombinant form of intrinsic human IL-1Ra. It works as an antagonist of IL-1R1, and it is able to inhibit both IL-1α and IL-1β. **c** Rilonacept is a recombinant fusion protein including the extracellular protein of human IL-1R1 and IL-1R3 fused with the Fc portion of human IgG1. It binds to both IL-1α and IL-1β with high affinity and has a long-term inhibitory effect. **d** Canakinumab and MABp1 are monoclonal antibodies against IL-1β and IL-1α, respectively. They bind to and neutralize their targets specifically

Several inhibitors of IL-1 signaling have been clinically approved (Fig. 1).

#### Anakinra

One is a recombinant human intrinsic IL-1 receptor antagonist (IL-1Ra), anakinra [147]. Anakinra is the pharmaceutical name of a recombinant form of intrinsic human IL-1Ra, a 17.2-kDa protein consisting of 153 amino acid residues. IL-1Ra was first reported in 1985 as a bioactive IL-1 inhibitor of 22–25 kDa in the supernatants of human monocyte culture, and it was independently identified as an IL-1 inhibitor from the urine of febrile patients [148, 149]. Anakinra was the first biological drug of a selective IL-1R1 antagonist to receive approval from the US Food and Drug Administration (FDA). Since anakinra is an IL-1 receptor antagonist, it can prevent the activity of both IL-1α and IL-1β by competitively blocking their binding to IL-1R1 and IL-1R2. Anakinra has been applied for a wide range of diseases including autoinflammatory diseases, non-cancer inflammatory diseases, and malignancies [150]. To date, no serious adverse effect of anakinra has been reported [151].

#### Rilonacept

Another is rilonacept (ril on’ a sept), a soluble decoy receptor (Fig. 1). Rilonacept is a recombinant fusion protein consisting of the extracellular portion of human IL-1R1 and IL-1R3 fused with the Fc portion of human IgG1 [152–154]. Rilonacept binds to both IL-1α and IL-1β with high affinity and inhibits the activity of both with a long-term inhibitory effect. Rilonacept was first approved by the FDA for the treatment of CAPS in 2008. Subcutaneous injection with a loading dose and a weekly injection of half the loading dose was administered [154]. There are no known severe adverse effects of rilonacept due to IL-1 signaling inhibition. These drugs could modulate the immune response. The most common side effects (> 10% of treated patients) are inflammation of the upper respiratory tract or sinuses, headache, and redness at the injection site [154].

#### Canakinumab

The third is canakinumab (Fig. 1). Canakinumab, a specific human monoclonal IgG1 antibody targeting IL-1β, is intravenously or subcutaneously infused to neutralize the bioactivity of human IL-1β [155, 156]. Canakinumab does not react with IL-1α or IL-1R1. Therefore, canakinumab is a more specific inhibitor of IL-1β, expected to have no effect on IL-1α-dependent host defense [154]. Early clinical trials established the administration of canakinumab every 2 weeks as safe and effective against several inflammatory diseases [155, 156].

#### MABp1

There are several agents currently undergoing clinical trials. IL-1α production is a very early step in the sterile inflammatory response at the center of the malignant phenotype that drives angiogenesis, tumor stromal remodeling, tumor invasiveness, metastasis, and cachexia [150, 157–159]. Thus, IL-1α may be a particularly important target for the treatment of cancer. A neutralizing true human IgG1κ monoclonal antibody specific for human IL-1α, MABp1, has been developed, and it was well-tolerated with no dose-limiting toxicities or immunogenicity [160, 161] (Fig. 1). MABp1 treatment for patients with advanced colorectal cancer in a randomized, double-blind, placebo-controlled, phase 3 study revealed that MABp1 improved clinical performance in patients with advanced colorectal cancer [161]. MABp1 is a promising treatment for patients with hidradenitis suppurativa not eligible for the anti-TNF-α antibody adalimumab [162].

#### Gevokizumab

Gevokizumab is an anti-IL-1β monoclonal antibody, IgG2, which improved glucose control and β-cell function in a diet-induced-obesity mouse model [163] and in the presence of IL-1β-driven inflammatory diseases [164].

#### LY2189102

LY2189102 is a humanized monoclonal antibody (IgG4) that binds to IL-1β to neutralize its activity. Its affinity is comparatively high (2.8 pmol/L). Previous clinical studies evaluated not only its safety and pharmacokinetics but also its effects on RA (NCT00380744). Weekly treatment of T2D patients with LY2189103 for 3 months resulted in modest reductions in glycated hemoglobin and blood glucose [165]. Population pharmacokinetics (PK) of LY2189102 were characterized using data from 79 T2D subjects (Study H9C-MC-BBDK) who received 13 weekly subcutaneous doses of LY2189102 (0.6, 18, and 180 mg) and 96 RA subjects (Study H9C-MC-BBDE) who received five weekly intravenous (IV) doses (0.02–2.5 mg/kg) [166]. No additional study has been reported.

#### AMG 108

AMG 108 is a fully human, IgG2 monoclonal antibody that binds to human IL-1R1, inhibiting the activity of IL-1α and IL-1β [167]. Patients with osteoarthritis received placebo or AMG 108 subcutaneously (SC, 75 or 300 mg) or intravenously (IV, 100 or 300 mg) once every 4 weeks for 12 weeks or received placebo or 300 mg AMG 108 SC, once every 4 weeks for 12 weeks; however, there was non-significant but numerically greater improvement in pain compared with the placebo group based on WOMAC pain scores [168]. AMG108 is now termed MEDI-8968 which has been studied in not only osteoarthritis, but also chronic obstructive pulmonary disease. In all cases, the benefit is limited [168, 169].

#### EBI-005

EBI-005 is a protein chimera of IL-1β and IL-1 receptor antagonists (IL-1Ra or anakinra). EBI-005 binds to IL-1R1 and inhibits IL-1 signaling and has been studied for the treatment of ocular surface inflammatory diseases [170].

#### VX-765

Since IL-1β is known to be processed and activated by caspase-1, caspase-1 could be an indirect target for IL-1β signaling. To examine this, the highly selective caspase-1 inhibitor VX-765 was applied to a rat model of myocardial infarction (MI) and mouse model of AD [171, 172].

### Applications of IL-1 blockade for diseases

#### For autoinflammatory diseases

The recombinant human IL-1-receptor antagonist anakinra is markedly effective against CAPS such as MWS, FCAS, and NOMID/CINCA. Weekly rilonacept treatment markedly improved the clinical symptoms of CAPS and normalized the levels of SAA in those at risk of developing amyloidosis [90, 153, 173, 174]. In several case reports of patients with FMF, anti-IL-1 treatment with anakinra, canakinumab, or rilonacept in colchicine-resistant patients was highly effective [175–178]. It was also reported that there was a rapid and lasting response of pyoderma gangrenosum to targeted treatment with anakinra in a patient with PAPA syndrome [179]. Anakinra and canakinumab therapies were also reported to be effective in patients with MKD/HIDS [180]. In the case of TRAPS, although TNF-α is considered to be mainly involved in clinical manifestations, marked improvement following IL-1β blockade occurred [112, 181]. An open-label, phase II study was reported whereby 19 patients with active recurrent or chronic TRAPS (19/20, 95%; 95% CI 75.1% to 99.9%) achieved the primary efficacy endpoint. Canakinumab treatment for TRAPS rapidly improved the median time to clinical remission to 4 days (95% CI 3 to 8 days) [182]. Skin findings also promptly improved upon anakinra treatment in a patient with DIRA [183]. Monotherapy involving canakinumab for the treatment of FMF has been reported [184]. A nationwide report on IL-1 treatment of patients with FMF revealed that 172 FMF patients (83 [48%] female; mean age, 36.2 years [range, 18–68]) were included; the mean age at onset was 12.6 years (range, 1–48), and the mean colchicine dose was 1.7 mg/day (range, 0.5–4.0). Anakinra was administered to 151 patients, and canakinumab was administered to 21 patients. Anti-IL-1 treatment was used in 84% of colchicine-resistant patients and 12% of amyloidosis patients. During the mean of 19.6 months of treatment (range, 6–98), the attack frequency per year was significantly decreased (from 16.8 to 2.4; *P* < 0.001), and symptoms of 42.1% of colchicine-resistant patients with FMF were ameliorated. In this study, the complete remission rate was 40% and inefficacy rate was 8% in patients treated with anakinra, whereas the complete remission rate was 65% and inefficacy rate was 6% for patients treated with canakinumab [185]. Although the response rates were not significant (*P* = 0.144 and χ2 = 3.872606) in the study above [185], in our opinion, long-acting canakinumab may be more efficacious than anakinra, considering the necessity of daily subcutaneous anakinra injection because of its short half-time clearance of less than 12 h [185].

#### For miscellaneous autoinflammatory diseases

There are suspected etiologies of autoinflammatory disorders, but all lack a known genetic basis. In patients with adult-onset Still’s disease (AOSD), anakinra monotherapy is significantly effective and has become the standard therapy, especially in prednisone-resistant patients. Commercially available anti-IL-1 agents (anakinra/Kineret®, canakinumab/Ilaris®, or rilonacept/Arcalyst®) for patients with treatment-resistant AOSD are effective. Canakinumab and anakinra were also effective for patients with Schnitzler syndrome, an adult-onset autoinflammatory disease characterized by focal urticaria and systemic inflammation including fever with bone and muscle pain, in the first placebo-controlled study, and several clinical trials are currently ongoing [186–189].

#### For autoimmune diseases

IL-1 blockade therapy using anakinra is successful in patients with psoriatic arthritis, ankylosing spondylitis, and RA. On the other hand, its efficacy and safety are insufficient, precluding use for patients with systemic lupus erythematosus or Sjögren syndrome, and IL-1β inhibition using canakinumab had no effect on the decline in β-cell function after diabetes onset in patients with type 1 diabetes mellitus resulting from autoimmune-mediated β-cell loss [190–194]. As for RA, the enhanced release of other proinflammatory cytokines such as TNF-α and IL-6 plays important roles in the inflamed synovium of RA patients [195]. In patients for whom TNF-α blockers are contraindicated, anakinra is effective in controlling the disease activity of RA and has been licensed for treatment at a dose of 100 mg/day by subcutaneous injection every day [196, 197]. Compared with anakinra, TNF inhibitors, such as the anti-TNF-α monoclonal antibody infliximab, or etanercept that fuse the TNF receptor to the end of the IgG1 antibody, dominate the field of biologics for RA because of the sense of well-being experienced by patients within hours of treatment [198]. Tocilizumab, a humanized anti-IL-6 receptor (IL-6R) monoclonal antibody, has also been shown to improve the symptoms of patients with RA [199]. However, those agents are associated with the risk of reactivating bacterial pathogens such as tuberculosis (TB) and malignancies [197]. Notably, no cases of TB reactivation were reported in 7964 RA patients after anakinra treatment, whereas 8 cases of TB reactivation were reported in 10,281 RA patients after tocilizumab treatment, and 7 and 10 cases of TB reactivation were reported in 2626 and 3167 RA patients after TNF-inhibitor treatment with golimumab and certolizumab pegol, respectively. This suggests the low risk of TB reactivation in RA patients treated with anti-IL-1 therapy [197].

#### For infectious diseases

Anakinra is safe and may be associated with a dose-related survival advantage in patients with septic shock syndrome complicated by acute respiratory distress syndrome, disseminated intravascular coagulation, and renal dysfunction, and subsequent secondary hemophagocytic lymphohistiocytosis (HLH), or macrophage-activating syndrome (MAS) [200, 201]. For sepsis with MAS, anakinra treatment led to significant improvements in hepatobiliary dysfunction and disseminated intravascular coagulation in patients (65.4% anakinra vs. 35.3% placebo) and the 28-day survival rate, with the hazard ratio for death being 0.28 (0.11–0.71; *p* = 0.0071) for the treatment group on Cox regression. The data included 763 adults in the original study cohort, randomized to receive either anakinra or placebo [202].

#### For metabolic syndromes

IL-1 inhibition by anakinra, rilonacept, or canakinumab is efficacious for gout patients [203]. IL-1 plays a role in the progression of atherosclerosis as well [204]. In patients with a history of MI, canakinumab significantly reduced the high-sensitive serum CRP concentration from the baseline, as compared with a placebo, without affecting the LDL-cholesterol concentration. A 150-mg dose of canakinumab resulted in a significantly reduced risk of recurrent cardiovascular events compared with a placebo [205]. The inhibition of IL-1 with anakinra improved glycemia and the pancreatic β-cell function and reduced systemic inflammation [205]. Although IL-1β inhibition with canakinumab had similar effects on major cardiovascular events among those with and without diabetes, treatment over a median period of 3.7 years did not reduce incident diabetes [206]. The blockade of IL-1 with anakinra improved glycemia and the β-cell secretory function and reduced markers of systemic inflammation [104]. Anakinra also prevented transthyretin extracellular deposition in the sciatic nerve in a familial amyloidotic polyneuropathy mouse model [207].

#### For ischemic diseases

During ischemic disease, such as MI or cerebral infarction, or tissue injury, cell death by necrosis takes place and the IL-1α precursor is released in sterile inflammation [208]. When there is no time for the synthesis of IL-1α, IL-1α is ready to function as soon as it leaves the dying cell in the first few hours following tissue ischemia or injury [209]. In fact, animal studies showed that the inhibition of IL-1 is effective in limiting atherosclerosis and cardiovascular events and improving the symptoms of acute MI and ischemic stroke [210, 211]. Two pilot studies of IL-1 inhibition in ST-segment elevation MI revealed a reduced acute inflammatory response and favorable overall performance at the 3-month follow-up [212].

#### For malignant diseases

IL-1β is thought to play an important role in cancer invasiveness, progression, and metastases via inflammation in the tumor microenvironment. A further analysis in the Canakinumab Anti-inflammatory Thrombosis Outcomes Study (CANTOS), a randomized trial of the role of inhibition of IL-1β in atherosclerosis, revealed that anti-inflammatory therapy with canakinumab targeting the IL-1β innate immunity pathway could significantly reduce lung cancer mortality [213]. Moreover, treatment of patients with metastatic breast cancer-related with anakinra eliminates a systemic transcriptional signature of IL-1-associated inflammation in blood cells. The inflammatory signature in primary breast cancers identifies a subset of patients that could potentially benefit from IL-1β-targeted therapies [129].

#### Safety profiles

**Fig. 2:**
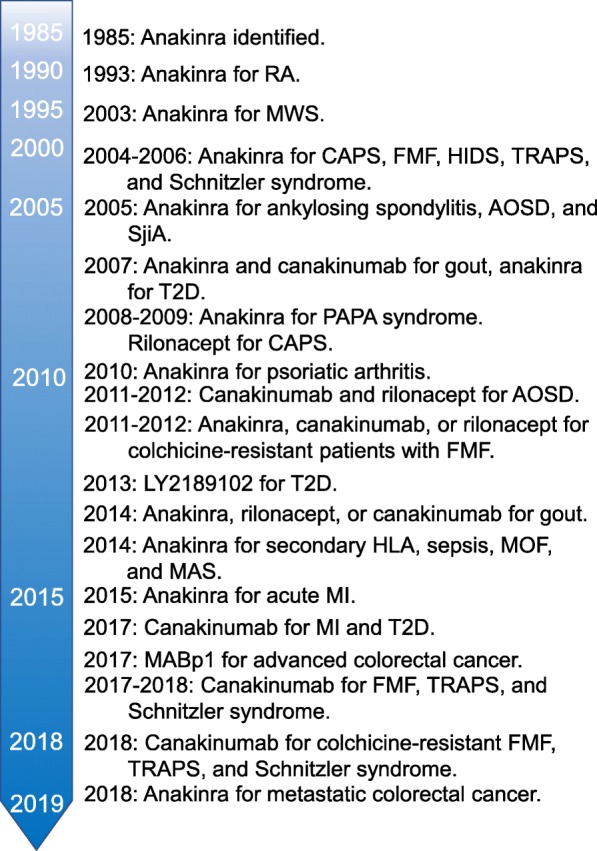
Timeline of anti-IL-1 therapy as described in the text. RA, rheumatoid arthritis; MWS, Muckle–Wells syndrome; CAPS, cryopyrin-associated periodic syndrome; FMF, familial Mediterranean fever; HIDS, hyper-IgD syndrome; TRAPS, TNF-receptor-1-associated periodic syndrome, AOSD, adult-onset Still’s disease; SjiA, systemic juvenile; T2D, type 2 diabetes mellitus; HLH, hemophagocytic lymphohistiocytosis; MOF, multiple organ failure; MAS, macrophage-activating syndrome; MI, myocardial infarction

Safety profiles of both anakinra and canakinumab were reported [214]. In this study, several clinical and therapeutic data on patients treated with either anakinra or canakinumab were retrospectively analyzed. Four hundred and seventy-five patients participated; anakinra and canakinumab were prescribed in 421 and 105 treatment courses, respectively. Eighty-nine adverse events were recorded; 13 (14.61%) were classified as serious adverse events during a mean follow-up of 24.39 ± 27.04 months. [214]. In addition, anakinra is applied to metastatic cancer. A trial involving metastatic colorectal cancer patients showed significantly increased survival when anakinra was added to standard chemotherapy for colorectal cancer and patients with HER2-negative breast cancer [129, 215]. The IL-1 blockade will reduce IL1-driven inflammation and immunosuppression that may contribute to the tumor metastatic microenvironment [216]. The timeline of therapeutics is summarized in Fig. 2.

## Conclusions

We described IL-1 as an important cytokine for not only inflammation related to cell injury but also homeostasis of cells, tissues, and organs in view of the general pathology. In addition, we also described recent expanding IL-1 signal-targeting for the treatment of diseases. Once the balance of IL-1 signaling is disrupted, it may markedly contribute to the pathogenesis of not only inflammatory disease, but also malignancies. IL-1-targeted biologics have been expanding, as there are no known serious adverse effects such as lymphoproliferative disorder or virus reactivation like TNF or IL-6-targeting therapies. Therefore, IL-1 is expected to become an attractive molecular target to treat a wide range of diseases such as autoinflammatory diseases, autoimmune diseases, infectious disease, metabolic syndromes, ischemic diseases, and malignant tumors [217, 218] (Table 2).

**Table 2:** Effective anti-IL-1 therapy for inflammatory diseases

Autoinflammatory diseases:	
Cryopyrin-associated periodic syndrome (CAPS) [87, 88]	
Familial Mediterranean fever (FMF) [95]	
Pyogenic arthritis, pyoderma gangrenosum and acne syndrome (PAPA) [96]	
NLRP12 autoinflammatory syndrome [97]	
Tumor necrosis factor receptor-1-associated syndrome (TRAPS) [100]	
Hyperimmunoglobulinemia D and periodic fever syndrome (HIDS)/mevalonate kinase deficiency (MKD) [180]	
Deficiency of the interleukin-1-receptor antagonist (DIRA) [183]	
Autoimmune diseases:	
Psoriatic arthritis [191]	
Ankylosing spondylitis [192]	
Rheumatoid arthritis (RA) [196]	
Metabolic syndrome:	
Gout [203]	
Atherosclerosis [204]	
Type 2 diabetes mellitus (T2D) [204]	
Amyloidosis [207]	
Neurodegenerative disease:	
Alzheimer’s disease (AD) [111]	
Infections and inflammatory responses:	
Septic shock syndrome [199]	
Acute respiratory distress syndrome (ARDS) [199]	
Disseminated intravascular coagulation (DIC) [199]	
Hemophagocytic lymphohistiocytosis (HLH) [200]	
Macrophage-activating syndrome (MAS) [200]	
Ischemic diseases:	
Myocardial infarction (MI) [209]	
Stroke [209]	
Malignant rumor:	
Breast cancer [129]	

## Abbreviations

AD: Alzheimer’s disease
AOSD: Adult-onset Still’s disease
APC: Antigen-presenting cell
CANTOS: Canakinumab Anti-inflammatory thrombosis Outcomes Study
CAPS: Cryopyrin-associated periodic syndrome
CARD: Caspase-recruitment domain
CINCA: Chronic infantile neurologic, cutaneous, and arthritis
CLR: C-type lectin receptor
CPPD: Calcium pyrophosphate dihydrate
CTL: Cytotoxic T lymphocyte
DAMP: Damage-associated molecular pattern
DC: Dendritic cell
DIRA: Deficiency of the IL-1-receptor antagonist
ERK: Extracellular signal-regulated kinase
FCAS: Familial cold autoinflammatory syndrome
FDA: Food and Drug Administration
FMF: Familial Mediterranean fever
GSDMD: Gasdermin-D
HIDS: Hyperimmunoglobulinemia D and periodic fever syndrome
HLH: Hemophagocytic lymphohistiocytosis
IAPP: Islet amyloid polypeptide
ICE: IL-1β-converting enzyme
IFN: Interferon
IKK: Iκ-B kinase
IL: Interleukin
IL-6 receptor: IL-6R
IL-18BP: Interleukin-18 binding protein
IL-1R1: IL-1 type 1 receptor
IL-1R2: IL-1 type 2 receptor
IL-1R3: IL-1 type 3 receptor
IL-1Ra: IL-1 receptor antagonist
IRAK: IL-1 receptor-associated kinase
JNK: c-Jun N-terminal kinase
LAF: Lymphocyte-activating factor
LPS: Lipopolysaccharide
MAPK: Mitogen-activated protein kinase
MAS: Macrophage activation syndrome
MDP: Muramyl dipeptide
MDSC: Myeloid-derived suppressor cell
MKD: Mevalonate kinase deficiency
MSU: Monosodium urate
MWS: Muckle–Wells syndrome
MyD88: Myeloid differentiation primary response protein 88
NEMO: NF-κB essential modulator
NK: Natural killer
NLR: NOD-like receptor
NLS: Nuclear localization sequence
NOMID: Neonatal-onset multisystem inflammatory disease
PAMP: Pathogen-associated molecular pattern
PAPA: Pyogenic arthritis, pyoderma gangrenosum, and acne
PAPASH: Pyogenic arthritis, acne, pyoderma gangrenosum and suppurative hidradenitis
PASH: Pyoderma gangrenosum, acne, and suppurative hidradenitis
PRR: Pattern recognition receptor
PYD: Pyrin-domain
RA: Rheumatoid arthritis
RLR: RIG-I-like receptor
ROS: Reactive oxygen species
T2D: Type 2 diabetes mellitus
TAM: Tumor-associated macrophage
TB: Tuberculosis
Th1: T-helper cell type 1
TIR: Toll-IL-1 receptor
TLR: Toll-like receptor
TNF: Tumor necrosis factor
TRAF: TNF receptor-associated factor
TRAPS: TNF-receptor-1-associated syndrome
Treg: Regulatory T cell
VEGF: Vascular endothelial growth factor
